# A Dynamic Model of Rescuer Parameters for Optimizing Blood Gas Delivery during Cardiopulmonary Resuscitation

**DOI:** 10.1155/2018/3569346

**Published:** 2018-11-29

**Authors:** Ali Jalali, Allan F. Simpao, Jorge A. Gálvez, Robert A. Berg, Vinay M. Nadkarni, Chandrasekhar Nataraj

**Affiliations:** ^1^Health Informatics Core, Johns Hopkins All Children's Hospital, 501 6th Avenue South, St. Petersburg, FL 33701, USA; ^2^Department of Anesthesiology & Critical Care Medicine, Children's Hospital of Philadelphia, Perelman School of Medicine at the University of Pennsylvania, 3401 Civic Center Blvd., Philadelphia, PA 19104, USA; ^3^Villanova Center for Analytics of Dynamic Systems, Department of Mechanical Engineering, Villanova University, Villanova, PA 19085, USA

## Abstract

**Introduction:**

The quality of cardiopulmonary resuscitation (CPR) has been shown to impact patient outcomes. However, post-CPR morbidity and mortality remain high, and CPR optimization is an area of active research. One approach to optimizing CPR involves establishing reliable CPR performance measures and then modifying CPR parameters, such as compressions and ventilator breaths, to enhance these measures. We aimed to define a reliable CPR performance measure, optimize the CPR performance based on the defined measure and design a dynamically optimized scheme that varies CPR parameters to optimize CPR performance.

**Materials and Methods:**

We selected total blood gas delivery (systemic oxygen delivery and carbon dioxide delivery to the lungs) as an objective function for maximization. CPR parameters were divided into three categories: rescuer dependent, patient dependent, and constant parameters. Two optimization schemes were developed using simulated annealing method: a global optimization scheme and a sequential optimization scheme.

**Results and Discussion:**

Variations of CPR parameters over CPR sequences (cycles) were analyzed. Across all patient groups, the sequential optimization scheme resulted in significant enhancement in the effectiveness of the CPR procedure when compared to the global optimization scheme.

**Conclusions:**

Our study illustrates the potential benefit of considering dynamic changes in rescuer-dependent parameters during CPR in order to improve performance. The advantage of the sequential optimization technique stemmed from its dynamically adapting effect. Our CPR optimization findings suggest that as CPR progresses, the compression to ventilation ratio should decrease, and the sequential optimization technique can potentially improve CPR performance. Validation *in vivo* is needed before implementing these changes in actual practice.

## 1. Introduction

Cardiopulmonary resuscitation (CPR) involves delivering chest compressions and positive pressure ventilation to cardiac arrest victims to maintain circulatory blood flow and oxygen delivery [1]. Studies have shown that the quality of CPR can impact post-CPR outcomes and survival [2–7]. CPR optimization remains an active topic of resuscitation research because survival rates for post-CPR hospital inpatients remain low [8]. Various approaches have been proposed to modify and optimize the CPR process. Simultaneous ventilation and compression as well as interposed abdominal compression are two examples of CPR modification [9–11].

Another approach to optimizing CPR involves defining and establishing reliable, appropriate CPR performance measures and then modifying CPR parameters to enhance these measures. Brain ischemia is a primary contributor to postarrest morbidity; thus, oxygen delivery and carbon dioxide elimination (end-tidal carbon dioxide [ETCO_2_]) have been established as important factors for measuring CPR performance [12, 13]. Other performance measures include mean coronary perfusion pressure (CPP), nitric oxide (NO), and balance of systemic and pulmonary perfusion with ventilation [14–17]. The CPR compression to ventilation ratio has been shown to be a CPR parameter that influences postarrest outcomes [18–21]. Another important CPR parameter is ventilation time, which is defined as the pause for ventilation between compressions. Experimental models have been used to assess the effects of ventilation time on coronary perfusion pressure [22].

In this study, we aimed to define a reliable CPR performance measure, optimize the quality of CPR based on the defined performance measure, and design a dynamically optimized scheme that varies CPR parameters to optimize CPR performance.

## 2. Materials and Methods

The Institutional Review Board at the Children's Hospital of Philadelphia approved this study.

### 2.1. Model-Based CPR Optimization

The combination of systemic oxygen delivery and carbon dioxide elimination at the lungs was selected as an objective function for maximization. Delivery of systemic carbon dioxide to the lung for elimination is associated with partial pressure of end-tidal carbon dioxide PETCO_2_ and consequently has been correlated with CPR outcome [23]. Maximizing the defined objective function will result in improving CPR performance by (i) ensuring sufficient oxygen delivery to vital organs and (ii) preventing carbon dioxide from accumulating in the body.

### 2.2. Calculating Blood Gas Delivery

During CPR, the blood gas (oxygen and carbon dioxide) delivery to any organ in the body can be estimated using the following simplified equation [24]:

(1) $$\begin{array}{c} D_{\text{BG}}=\bar{Q}\Delta C_{\text{BG}}, \end{array}$$

where $\bar{Q}$ is the mean blood flow during CPR, ∆*C* is the change in the blood gas concentration, and subscript BG refers to blood gas. Mean blood flow or $\bar{Q}$ during CPR is

(2) $$\begin{array}{c} \bar{Q}=Q_{\max}\frac{x}{\left(T/t\right)+x}, \end{array}$$

where *T* is the average artificial ventilation time, *t* is the time for one full compression, (1/*t*) is the compression speed, and *x* is the compression to ventilation ratio [16]. For systemic oxygen delivery, *Q*_max_ is the maximum systemic blood flow *Q*_*s*_, and for carbon dioxide delivery to the lungs, *Q*_max_ is the maximum pulmonary blood flow *Q*_*p*_. On average, we assume that systemic blood flow is equal to pulmonary blood flow; hence, $\bar{Q}$ is equal for both the cases of systemic oxygen delivery and carbon dioxide delivery to the lungs.

To calculate ∆CO_2_ and ∆*C*_CO2_, we need to start with pulmonary gas exchange equations presented in [17]. The presented model assumes a steady-state condition as well as continuous ventilation and perfusion. The fraction of alveolar gas (*f*_*A*_) is equal to end-tidal gas,

(3) $$\begin{array}{c} f_{{\text{ET}}_{{\text{CO}}_{2}}}=f_{A_{{\text{CO}}_{2}}},\\ f_{{\text{ET}}_{{\text{O}}_{2}}}=f_{A_{{\text{O}}_{2}}}. \end{array}$$

In the steady-state condition, the oxygen and carbon dioxide balance in the lungs can be expressed using the following equations:

(4) $$\begin{array}{c} {\left({\dot{Q}}_{{\text{O}}_{2}}\right)}_{\text{in}}={\left({\dot{Q}}_{{\text{O}}_{2}}\right)}_{\text{out}}+\bar{Q}p_{{\text{O}}_{2}}f_{A_{{\text{O}}_{2}}},\\ {\left({\dot{Q}}_{{\text{CO}}_{2}}\right)}_{\text{out}}={\left({\dot{Q}}_{{\text{CO}}_{2}}\right)}_{\text{in}}+\bar{Q}s_{{\text{CO}}_{2}}f_{A_{{\text{CO}}_{2}}}, \end{array}$$

where subscript in refers to tracheal inflow, subscript out refers to tracheal outflow, and *s*_O_2__ and *s*_CO_2__ are slopes of oxygen and carbon dioxide dissociation curves, respectively. In CPR-related parameters,

(5) $$\begin{array}{c} \left(v_{t}-v_{d}\right)Rf_{I_{{\text{O}}_{2}}}=\left(v_{t}-v_{d}\right)Rf_{A_{{\text{O}}_{2}}}+{\bar{Q}}_{s}s_{{\text{O}}_{2}}f_{A_{{\text{O}}_{2}}},\\ \left(v_{t}-v_{d}\right)Rf_{A_{{\text{CO}}_{2}}}=\left(v_{t}-v_{d}\right)Rf_{I_{{\text{CO}}_{2}}}+{\bar{Q}}_{p}s_{{\text{CO}}_{2}}f_{A_{{\text{CO}}_{2}}}, \end{array}$$

where *R* is the average ventilation rate, *v*_*t*_ is the tidal volume, and *v*_*d*_ is the dead-space volume. Solving for alveolar fractions, we get

(6) $$\begin{array}{c} f_{A_{{\text{O}}_{2}}}=\frac{\left(v_{t}-v_{d}\right)Rf_{I_{{\text{O}}_{2}}}}{\left(v_{t}-v_{d}\right)R+{\bar{Q}}_{s}s_{{\text{O}}_{2}}},\\ f_{A_{{\text{CO}}_{2}}}=\frac{\left(v_{t}-v_{d}\right)Rf_{I_{{\text{CO}}_{2}}}}{\left(v_{t}-v_{d}\right)R-{\bar{Q}}_{p}s_{{\text{CO}}_{2}}}. \end{array}$$

Since changes in concentrations are small, they are in the linear range of dissociation curves, or in mathematical terms:

(7) $$\begin{array}{c} \Delta C_{{\text{O}}_{2}}=s_{{\text{O}}_{2}}f_{A_{{\text{O}}_{2}}},\\ \Delta C_{{\text{CO}}_{2}}=s_{{\text{CO}}_{2}}f_{A_{{\text{CO}}_{2}}}. \end{array}$$

Finally, combining the above equations will result in mathematical expressions for systemic oxygen delivery and carbon dioxide delivery:

(8) $$\begin{array}{c} D_{{\text{O}}_{2}}=Q_{s,\max}\frac{s_{{\text{O}}_{2}}x\left(v_{t}-v_{d}\right)f_{I_{{\text{O}}_{2}}}}{\left(\left(T/t\right)+x\right)\left(\left(v_{t}-v_{d}\right)+tx{\bar{Q}}_{s,\max}s_{{\text{O}}_{2}}\right)},\\ D_{{\text{CO}}_{2}}=Q_{p,\max}\frac{s_{{\text{O}}_{2}}x\left(v_{t}-v_{d}\right)f_{I_{{\text{O}}_{2}}}}{\left(\left(T/t\right)+x\right)\left(\left(v_{t}-v_{d}\right)-tx{\bar{Q}}_{p,\max}s_{{\text{CO}}_{2}}\right)}, \end{array}$$

where *Q*_*s,*max_ and *Q*_*p,*max_ are maximum systemic and pulmonary blood flow, respectively. In steady-state CPR condition, *Q*_*s,*max_ and *Q*_*p,*max_ are equal to ensure balanced blood flow to the body and the lungs. The objective function we sought to maximize was the total blood gas delivery, defined as the summation of carbon dioxide delivery to the lung and systemic oxygen delivery. It should be noted that a differentially weighted sum of the two deliveries might also be a possible criterion.

(9) $$\begin{array}{c} D_{\text{Total}}=D_{{\text{O}}_{2}}+D_{{\text{CO}}_{2}}. \end{array}$$

### 2.3. Optimization

In the next step, CPR parameters were divided into three categories: rescuer dependent, patient dependent, and constant parameters. Rescuer-dependent parameters are parameters that depend on rescuer performance, such as *f*_*I*_CO_2___. Patient-dependent parameters are parameters that vary from patient to patient, such as *Q*_max_. Constant parameters are fixed parameters, such as *s*_CO_2__. In the optimization procedure, we varied the rescuer-dependent parameters for each patient (i.e., with constant patient-dependent parameters) over the entire range of patients. The described parameters and their values are listed in Table 1.

Next, two different optimization schemes were developed: a global optimization scheme and a sequential optimization scheme. Both of the methods used simulated annealing as the optimization technique. Simulated annealing is a computational intelligence technique that aims to solve combinatorial optimization problems to minimize the defined cost function which is characterized by a large number of solutions [25]. The simulated annealing method has been widely applied to problems in the fields of engineering, science, and biomedical research [26–29].

Unlike most of the optimization techniques which could be considered analogous to rapid cooling methods, simulated annealing is analogous to a process of slowly cooling a physical system by providing ample time in order to obtain states with globally minimum energy [30]. As a result, simulated annealing is able to find solutions near a global minimum for very large optimization problems. To define the simulated annealing, we first defined the energy of the system and determined how it related to the temperature of the system. The Helmholtz free energy of a physical system represented by function *f* at a given temperature *T* is defined by the following equation:

(10) $$\begin{array}{c} E_{\emptyset }=\underset{\emptyset }{\sum }p_{\emptyset }E_{\emptyset }+T\underset{\emptyset }{\sum }p_{\emptyset }\;\log\;p_{\emptyset }, \end{array}$$

where *p*_*ϕ*_ is the probability of occurrence of state *ϕ*, which could be calculated using the following equation:

(11) $$\begin{array}{c} p_{\emptyset }=\frac{\exp\left(-f\left(\emptyset \right)/t_{k}\right)}{\sum_{\emptyset^{i}\in \Phi }\exp\left(-f\left(\emptyset^{i}\right)/t_{k}\right)}, \end{array}$$

Φ is a set of all possible states of the system. This equation will result in the conclusion that low energy ordered states are strongly favored at low temperatures.

#### 2.3.1. Global Optimization

First, we considered global optimization. In this method, for each patient group, i.e., a fixed *Q*_max_, we sought to maximize the total blood gas delivery that is expressed by Equation (1). We used rescuer-dependent parameters as a set of free parameters of the model for a range of fixed *Q*_max_ to find the global maximum for total delivery. Instead of a set of fixed values for all of the patients as recommended by American Heart Association CPR guidelines, the proposed global optimization method tried to find the set of parameters for each patient group that resulted in maximum blood gas delivery.

#### 2.3.2. Sequential Optimization

This method involved applying a sequential optimization scheme to actively vary the CPR parameters in order to maximize the defined objective function and thereby improve CPR performance. In this scheme, instead of finding the global maximum of the objective function for each patient group, at each CPR sequence, the rescuer-dependent parameters were varied based on a predefined protocol to find the global maximum of blood gas delivery. We defined a CPR sequence as one cycle of compressions and positive pressure ventilation. During each CPR sequence, a compression to ventilation ratio that maximized the total delivery was found, and then the CPR was performed. Total delivery was then maximized again by varying other rescuer-dependent parameters. These new parameters were used as the starting point of the next CPR sequence, and this procedure was continued until reaching the maximum total delivery for each group.

The steps of the sequential optimization algorithm can hence be summarized as follows:

1. Start with nominal values.
2. Optimize the total delivery based on varying *x*.
3. Rescuer performs the CPR cycle.
4. Optimize the remaining rescuer parameters to maximize the oxygen delivery with a fixed *x*.
5. Go to Step 2 and repeat until objective function shown in Equation (9) reaches the maximum.

To simulate a wide range of patient groups, we varied *Q*_max_ in the range of 700 to 1100 mL blood per minute.

## 3. Results and Discussion

**Figure 1:**
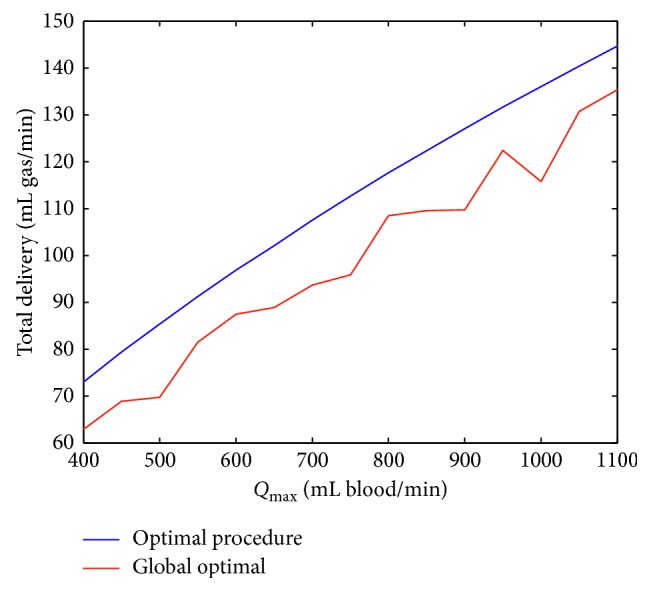
Results obtained from the two proposed schemes for optimizing total blood gas delivery during cardiopulmonary resuscitation. For systemic oxygen delivery, *Q*_max_ is the maximum systemic blood flow *Q*_*s*_, and for carbon dioxide delivery to the lungs, *Q*_max_ is the maximum pulmonary blood flow *Q*_*p*_.

Results obtained from two different optimization prospective are presented in Figure 1. Results show (1) that the proposed sequential algorithm is more effective in optimizing CPR performance, and (2) increasing *Q*_max_ will increase total blood gas delivery.

**Figure 2:**
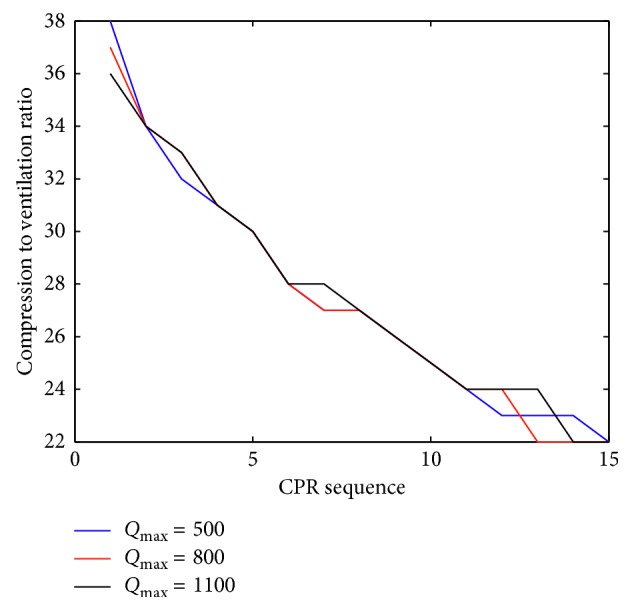
Plot of compression to ventilation ratio for three different *Q*_max_, (i.e., three different patient groups) during cardiopulmonary resuscitation (CPR).

Figure 1 shows that, in all the patient groups, the sequential optimization scheme will result in significant enhancement in the effectiveness of the CPR procedure. The advantage of the sequential optimization technique stemmed from its dynamically adapting effect. Compression to ventilation ratios for three different *Q*_max_ are presented in Figure 2.

Figure 2 shows the compression to ventilation ratio for three different *Q*_max_, (i.e., three different patient groups) during CPR. During initiation of CPR, the rescuer should place more emphasis on chest compressions than on ventilation (high compression to ventilation ratio) and gradually decrease the compression to ventilation ratio and carry out the positive pressure ventilation more frequently.

**Figure 3:**
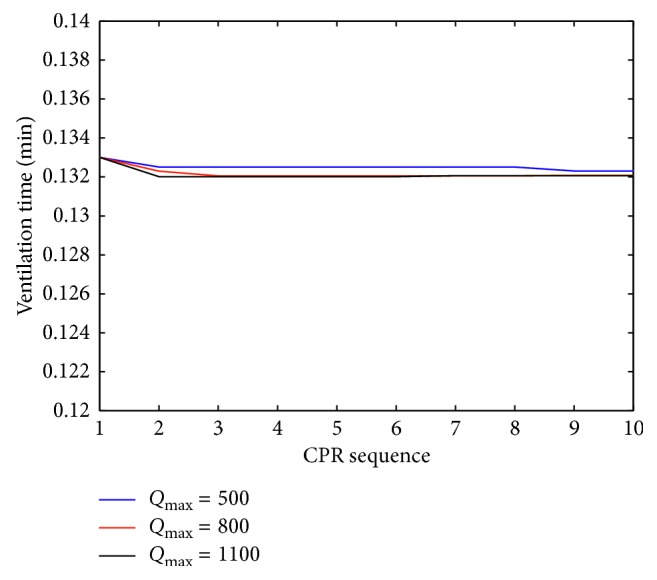
Plot of average ventilation time for three different *Q*_max_ values during cardiopulmonary resuscitation (CPR).

The results obtained for average ventilation time *T* for three different *Q*_max_ are presented in Figure 3. The plot shows that the ventilation time does not change over the time and remains constant for all patient groups.

**Figure 4:**
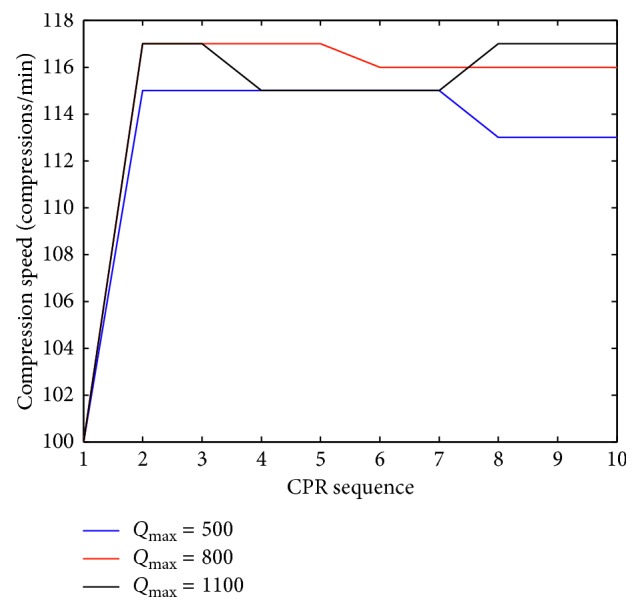
Plot of compression speed over time for three different *Q*_max_ values during cardiopulmonary resuscitation (CPR).

The results obtained for compression speed 1/*t* for three different *Q*_max_ are presented in Figure 4. This figure also shows that there should be interactive changes in the rescuer's performance with progression of the CPR. The plot shows that the compression speed should generally increase as time progresses.

## 4. Conclusions

In this study, we present an analysis of dynamic modeling of CPR parameters during CPR to improve CPR performance. While previous research efforts to optimize CPR have focused on finding the best compression to ventilation ratio and keeping the ratio constant during the CPR procedure, we propose a sequential optimization scheme to vary the rescuer-dependent parameters as needed during CPR sequences to optimize CPR performance.

Our results suggest that as CPR progresses, the compression to ventilation ratio should decrease over time to optimize performance. One possible explanation for this observation in our model is that when CPR typically commences, blood oxygen content is sufficient for satisfactory systemic delivery and the carbon dioxide level has only started to rise above normal levels. As CPR progresses, oxygen decreases in the blood and carbon dioxide starts to accumulate, so ventilation becomes increasingly important. Hence, our model suggests that the optimal compression to ventilation ratio should begin higher than is currently practiced (greater than 30 compressions per ventilation) and then decrease over time.

In this study, we followed to a large extent the methods as described in a conference paper [31]. In that earlier study, we used systemic oxygen delivery as a performance measure for optimization [31]. A model developed earlier by Babbs et al. [16] was used to calculate the oxygen delivery through CPR. The free parameters of the previous model that depended on rescuer performance were ventilation time, compression speed, tidal volume, and fraction of oxygen in the inspired air. Two different optimization problems were carried out. Results obtained from that study showed that the potential of the sequential optimization procedure to enhance the performance of the CPR, but the results of that study only recommended one drop in compression to ventilation ratio during dynamic phase. Promising results from that study encouraged us to explore the subject of dynamic variation of CPR parameters with a broader, more realistic objective function: total blood gas delivery.

There were several limitations to this study. First, we used one published systemic oxygen delivery model to develop analytical expressions for both systemic oxygen delivery and carbon dioxide delivery to the lungs [32], and we developed and analyzed only two approaches for optimization. Our findings are not necessarily reproducible using different physiological models and analytical approaches. Second, we selected parameter ranges for the optimization process based on expert knowledge and our experiences; others may find different parameter ranges more relevant to their practice population. In the next step, we plan to apply the sequential optimization method on a more complex model of chest that we developed to add systemic blood flow to the objective function for more comprehensive CPR optimization [33]. Lastly, our findings are based on mathematical modeling and have not been validated *in vivo*, and they should not be used to adjust current CPR practices until such validation has been completed.

In summary, our study illustrates the potential benefit of considering dynamic changes in rescuer-dependent parameters during CPR in order to improve performance. Validation in an animal model and an assessment of generalizability to real data sets are important future steps.

## Figures and Tables

**Table 1:** Parameter values and their respective ranges.

Parameter	Range	Unit
CPR parameters		
*T*	[0.132,0.134]	Min
*v*_*t*_	[600,1000]	mL
*t*	[0.008,0.012]	Min
*f*_*I*_O_2___	[0.13,0.19]	mL·O_2_/mL gas
*f*_*I*_CO_2___	[0.0002,0.0006]	mL·O_2_/mL gas

Patient specific parameter		
*Q*_max_	[400,1000]	mL/min

Constant parameters		
*v*_*d*_	150	mL
*s*_CO_2__	0.8	mL/mL
*s*_O_2__	1.5	mL/mL

The range for each parameter shows the lower and upper limit of variable changes during the optimization. Except for the constant parameters, parameter values change during the CPR delivery.

## Data Availability

The data used to support the findings of this study are available from the corresponding author upon request.
